# Macrophage-derived chemokine CCL22 and regulatory T cells in ovarian cancer patients

**DOI:** 10.1007/s13277-015-3133-8

**Published:** 2015-02-03

**Authors:** I. Wertel, J. Surówka, G. Polak, B. Barczyński, W. Bednarek, J. Jakubowicz-Gil, A. Bojarska-Junak, J. Kotarski

**Affiliations:** 1 I Chair and Department of Oncological Gynaecology and Gynaecology, Medical University of Lublin, Staszica 16, 20-081 Lublin, Poland; 2 Department of Comparative Anatomy and Anthropology, Maria Curie-Skłodowska University, Lublin, Poland; 3 Department of Clinical Immunology, Medical University of Lublin, Lublin, Poland

**Keywords:** CCL22, Peritoneal fluid, Regulatory T cells, Ovarian cancer

## Abstract

The study was undertaken to evaluate macrophage-derived chemokine (CCL22) levels in the peritoneal fluid (PF) and plasma of patients with ovarian cancer (*n* = 93) in relation to regulatory T cells (Tregs; *n* = 75). The peritoneal fluid CCL22 concentrations were significantly higher in epithelial ovarian cancer (EOC) patients than in patients with benign tumors-serous cystadenoma (*n* = 32). There was no difference in plasma levels of CCL22 in EOC patients compared with the non-cancer and healthy volunteers (*n* = 10). There were no significant differences in the plasma and PF CCL22 levels based on tumor grade. However, women with stage IV FIGO (International Federation of Gynecologists and Obstetricians) had significantly higher plasma CCL22 levels than patients with stages I and III. Women with stage I FIGO had significantly higher PF CCL22 levels than patients with stages II and III. Women with endometrioid cystadenocarcinoma had higher PF CCL22 levels than women with undifferentiated carcinoma. The percentage of tumor-infiltrating Tregs (11.06 %) was significantly higher compared to PF (3.05 %) and peripheral blood (PB) (2.01 %). Moreover, the percentage of Tregs was higher in the PF than in the PB of EOC patients. There were no significant differences in the PB, PF, and tumor-infiltrating Tregs percentage based on tumor stage, grade, or histology. Elevated levels of CCL22 found in the ascites could create a chemokine gradient aiding in Treg cells migration. Increased Tregs percentage in the local microenvironment of ovarian cancer might be an important mechanism of immunosuppression.

## Introduction

The development of epithelial ovarian cancer (EOC) is associated with several immunosuppressive elements, involving regulatory T cells (Tregs) activity in the tumor microenvironment. It was shown that infiltration of ovarian cancers by Tregs suppresses tumor-specific T cell immune response and contributes to the tumor growth in vivo [1]. It has also been documented that Tregs can inhibit the local immune response through both direct cell-to-cell contact and secretion of immunosuppressive cytokines: TGF-β and IL-10 [reviewed in 2].

Tregs are a heterogeneous CD4^+^ T cell subpopulation, which are characterized by coexpression of CD25 and other Treg characteristic markers, such as a glucocorticoid-induced tumor necrosis factor receptor (GITR), a cytotoxic T lymphocyte antigen-4 (CTLA-4), and forkhead box protein 3 (FoxP3), which is a highly specific and reliable marker set for primary human Tregs [3].

In 2001, Woo et al. [4] detected an increased percentage of Tregs in women with late stage ovarian cancer. Similarly, Curiel et al. [1] found large numbers of Tregs in malignant ascites and in the tumor mass of patients with EOC and showed a strong association of Tregs with poor survival. An independent study by Wolf et al. [5] confirmed that a high expression of the FoxP3 on Tregs is a negative prognostic factor for both progression-free and overall survival in EOC patients. In contrary to the previous studies, Leffers et al. [6] and Milne 2009 et al. [7] showed that tumor-infiltrating Tregs were an independent positive factor for disease-specific survival in EOC patients. Therefore, the data concerning the importance of Tregs in affecting ovarian cancer patients' outcome is controversial.

A few studies have demonstrated the importance of the macrophage-derived chemokine (CCL22) and its receptor CCR4 in the migration of Tregs [8, 1, 9, 10]. The macrophage-derived chemokine is produced constitutively by macrophages [11, 1], monocyte-derived dendritic cells [11, 1, 12, 13], activated natural killer (NK) cells [10], activated T cells [11, 14], and epithelial cells [15, 14]. Recently, it has been reported that several solid tumor cells, including ovarian [1], breast [10, 9, 16], prostate [17], gastric [18], and esophageal tumor cells [19], release CCL22.

CCL22 plays an important role in a variety of diseases, including allergic rhinitis [20], atopic dermatitis [21], and lymphoma [22]. The role of CCL22 in malignant diseases has not been investigated extensively. Li and co-workers [16] have demonstrated that breast tumor cell-derived CCL22 can serve as an independent prognostic predictor of breast cancer patient’s survival. CCL22 might be also important for the immunology of ovarian cancer. It influences migration of activated T cells [23], activated NK cells, monocytes, and monocyte-derived dendritic cells [11]. It was shown that CCL22 is also a chemoattractant for regulatory T cells [1].

In this study, we investigated the concentrations of CCL22 in the peritoneal fluid and plasma of patients with malignant and benign ovarian disease and assessed relationship between peritoneal fluid (PF) and peripheral blood (PB) regulatory T cells and CCL22. To our knowledge, there are no previous reports of the plasma and peritoneal fluid CCL22 and triple stained (CD4^+^CD25^high^FoxP3^+^) Tregs percentage in women with different stage, grade, and histological type of epithelial ovarian cancer.

## Materials and methods

### Patients

A total of 93 women with histologically confirmed ovarian cancer were enrolled in this study. Clinical stage was determined according to FIGO (*International Federation of Gynecologists and Obstetricians*) classification [24]. Tumors were graded and classified according to Shimizu-Silverberg grading system by two independent gynecological pathologists [25]. The clinical and pathological characteristics of the EOC patients are summarized in Table 1. None of the women received chemotherapy before surgery. The reference group consisted of 32 patients with benign tumors (histology: serous cystadenoma), with no evidence of malignancies or pelvic adhesions. The control group consisted of ten healthy peripheral blood donors. Informed consent was obtained from all individual participants included in the study. The study was approved by Lublin Medical University Ethics Committee.

**Table 1:** Patient’s characteristics

Parameters	Value
Patient age, *n* (min-max)	
Ovarian cancer	55 (24–89)
Serous cystadenoma	27 (18–76)
Histology, *n* (%)	
Serous cystadenocarcinoma (S)	34 (36.55)
Endometrioid cystadenocarcinoma (E)	24 (25.80)
Undifferentiated carcinoma (U)	23 (24.73)
Mucinous cystadenocarcinoma (M)	12 (12.90)
Serous cystadenoma	32 (100 %)
Grading, *n* (%)	
G II	35 (37.63)
G III	58 (62.36)
FIGO stage, *n* (%)	
I	9 (9.67)
II	14 (15.05)
III	54 (58.06)
IV	16 (17.20)

### Methods

Peripheral blood was collected in heparinized tubes. Peritoneal fluid specimens were obtained at the time of surgery. Plasma and PF samples were rendered cell-free by centrifugation at 1500 rpm for 10 min and stored at −80 °C before being tested by enzyme-linked immunosorbent assay (ELISA).

CCL22 concentrations in plasma and PF were determined by Immunoassay kit (Research and Diagnostic Systems, Minneapolis, Minnesota, USA) following the manufacturer’s protocol. Concentrations of CCL22 were calculated by interpolation from a standard curve. The sensitivity of the CCL22 ELISA was 62.5 pg/ml. All samples were assayed in duplicate.

### Cell preparation

PF and PB were taken into heparinized tubes (sodium heparin). In the non-cancer group, all visible PF was aspirated during surgical procedure from the anterior and posterior cul-de-sacs, under direct vision to avoid blood contamination. All women had venous blood samples collected before the surgical procedure. PF and PB mononuclear cells (*n* = 75) were isolated by density gradient centrifugation on Lymphoprep (Nycomed, Norway) for 25 min at 600 g at room temperature. Interface cells were collected and washed twice in phosphate-buffered saline (PBS).

Tumor-infiltrating lymphocytes (TILs) were harvested from 33 patients and isolated by discontinuous Ficoll gradient as described by Knutson et al. [26]. Briefly, the lymphocytes were separated from the tumor cells by centrifugation of the cell suspension on a two layer Ficoll gradient, 100 % layer on the bottom and 75 % layer on top. The isolated cells were frozen in storage medium (composition: 10 % dimetyl sulfoxide (Sigma-Aldrich, USA), 20 % human albumin (Baxter, Poland), and 70 % RPMI 1640 medium (PAA, Austria)) and kept in liquid nitrogen to the time of flow cytometry analysis of Tregs. The percentage of Tregs was determined using the Human Treg Flow Kit from BioLegend (San Diego, CA, USA) according to the manufacturer’s instructions.

T regulatory cells were evaluated via analysis of the surface expression of CD4 and CD25 antigens, as well as intracellular expression of FoxP3. The isolated cells were incubated with mAbs for surface staining such as anti-CD4 PE-Cy5 and anti-CD25 PE (BioLegend). We also evaluated expression of CCR4 receptor on Tregs using anti-CCR4 PE-Cy7 monoclonal antibody and relevant izotype control (BD Pharmingen). Next, cells were permeabilized and stained with anti-FoxP3 Alexa Fluor 488 and relevant isotype controls (BioLegend). After the intracellular staining, cells were washed and estimated by flow cytometry.

### Flow cytometric analysis

Flow cytometric analysis of stained samples was performed with a FacsCanto flow cytometer (Becton Dickinson, San Jose, California, USA). A total of 100,000 events were acquired and analyzed using FacsDiva software. Cell debris and dead cells were excluded from the analysis based on scatter signals. T regulatory cells were characterized as CD4^+^CD25^high^ expressing FoxP3^+^. Results are expressed as a percentage of Tregs among CD4^+^ T cells.

### Statistical analysis

Data were presented as medians with the interquartile ranges. The Wilcoxon paired test was used to compare the results in PF, PB, and tissue. The Mann-Whitney *U* test was applied to the results of statistical comparison between the studied groups. Spearman’s rank test was used to assess the relationship between concentrations of CCL22 and Tregs numbers. *p* value less than 0.05 was considered statistically significant.

## Results

### Concentration of CCL22 in the peritoneal fluid and plasma of women with ovarian cancer and serous cystadenoma and in the control group

The concentrations of CCL22 in the peritoneal fluid and plasma of patients with ovarian tumors and normal control group are presented in Table 2.

**Table 2:** Levels of CCL22 (pg/ml) in the plasma and peritoneal fluid (PF) of patients with ovarian tumors and normal donors

Group of patients	PLASMA		PF	
	Median	Interquartile range	Median	Interquartile range
Ovarian cancer (*n* = 93)	299.81	237.63-459.53	435.39^b^	328.65-607.91
Serous cystadenoma (*n* = 32)	360.94^a^	256.58-449.90	274.55	225.79-318.17
Normal donors (*n* = 10)	437.05	337.73-470.78		

^a^ *p* < 0.001 in relation to PF of patients with serous cystadenoma

^b^ *p* < 0.001 in relation to PF of patients with serous cystadenoma and in relation to plasma of EOC patients

CCL22 levels detected in the peritoneal fluid of women suffering from ovarian cancer were found to be significantly higher (*p* < 0.001) than those with benign ovarian disease.

CCL22 levels detected in the peritoneal fluid of women suffering from ovarian cancer were found to be significantly higher (*p* = 0.00002) than in the plasma. In contrary, in the group of patients with benign ovarian disease, the CCL22 levels were significantly higher in the plasma (*p* < 0.001) than in the peritoneal fluid (Table 2).

There was no significant difference in the plasma CCL22 levels among the ovarian cancer patients, the benign ovarian tumor patients, and the control group (Table 2).

### Concentration of CCL22 in patients with different stage, grade, and histologic type of ovarian cancer

**Fig. 1:**
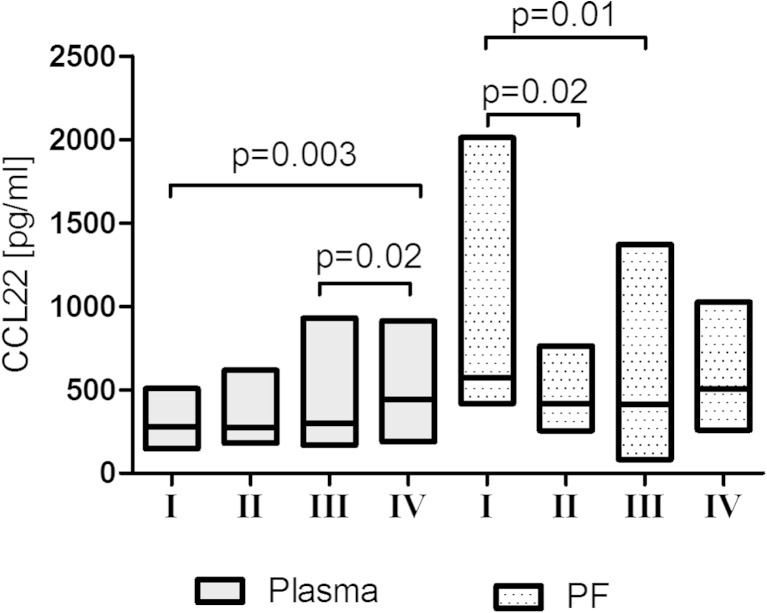
Levels of CCL22 (pg/ml) in the plasma and peritoneal fluid (PF) of patients with different FIGO stage of ovarian cancer [*line* at median; *floating bars* (min to max)]

**Fig. 2:**
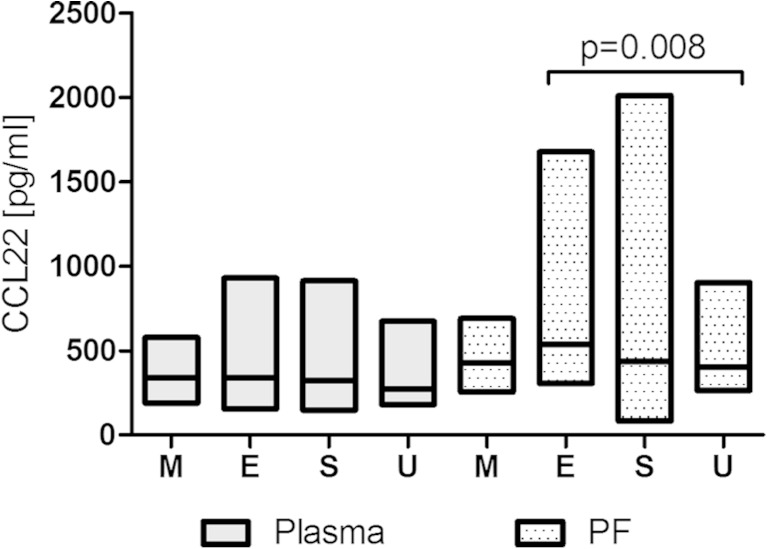
Levels of CCL22 (pg/ml) in the plasma and peritoneal fluid (PF) of patients with different histological type of ovarian cancer [*line* at median; *floating bars* (min to max)]

The PF CCL22 levels in patients with FIGO stage I of ovarian cancer were significantly higher than in women with FIGO stages II and III (*p* < 0.05; Fig. 1). Women with endometrioid cystadenocarcinoma had significantly higher (*p* < 0.05) PF CCL22 levels than patients with undifferentiated carcinoma (Fig. 2).

The plasma CCL22 levels in patients with FIGO stage IV of ovarian cancer was significantly higher than in women with FIGO stage I (*p* = 0.003) and FIGO stage III (*p* = 0.02) (Fig. 1). There were no significant differences in the plasma CCL22 levels in relation to histology (Fig. 2).

There were no significant differences in the plasma and PF CCL22 levels in relation to tumor grade (Table 3).

**Table 3:** Levels of CCL22 (pg/ml) in the plasma and peritoneal fluid (PF) of patients with ovarian cancer in relation to tumor grade

Group of patients	PLASMA		PF	
	Median	Interquartile range	Median	Interquartile range
G2 (*n* = 35)	278.67	205.12–420.01	443.82^a^	345.22–697.84
G3 (*n* = 58)	321.27	262.77–504.19	435.39^b^	314.60–597.12

^a^ *p* = 0.002 in relation to plasma of G2 patients

^b^ *p* = 0.003 in relation to plasma of G3 patients

### The percentage of Tregs in women with ovarian cancer and benign tumors

The percentage of tumor-infiltrating Tregs was significantly higher (11.06 %; interquartile ranges 6.87 to 19.28 %) compared to PF (3.05 %; interquartile ranges 1.08 to 4.99 %) and PB (2.01 %; interquartile ranges 1.09 to 3.45 %). Moreover, the percentage of Tregs was higher (*p* = 0.0004) in the PF than in the PB of EOC patients.

**Fig. 3:**
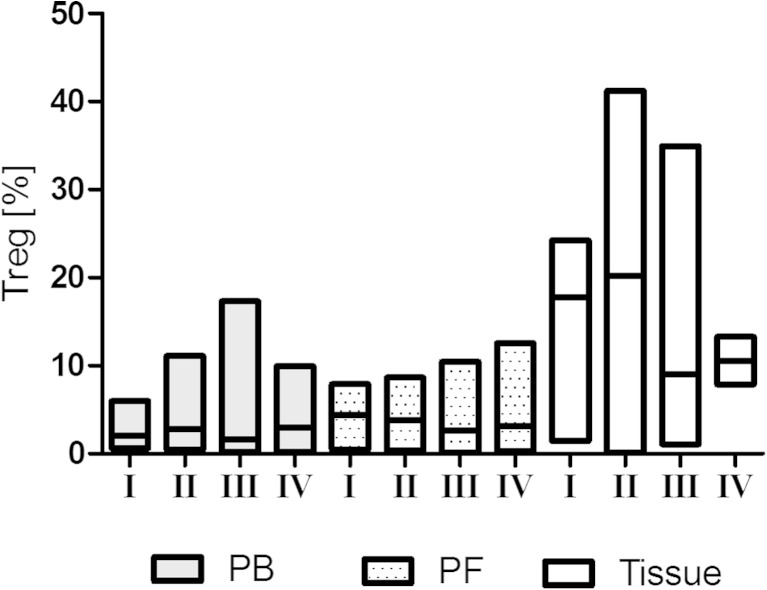
The percentage of PB, PF, and tumor-infiltrating Tregs in women with different FIGO stage of ovarian cancer [*line* at median; *floating bars* (min to max)]

There were no significant differences in the PB, PF, and tumor-infiltrating Tregs levels based on tumor stage (Fig. 3), grade, or histology (*p* > 0.05).

The percentage of Tregs in the PB of women with ovarian cancer (2.01 %; interquartile ranges 1.09 to 3.45 %) was higher than in patients with serous cystadenoma (0.94 %; interquartile ranges 0.52 to 2.68 %), but the difference was not statistically significant (*p* = 0.42).

### Relationship between concentration of CCL22 and Tregs in the PF and PB of EOC patients

**Fig. 4:**
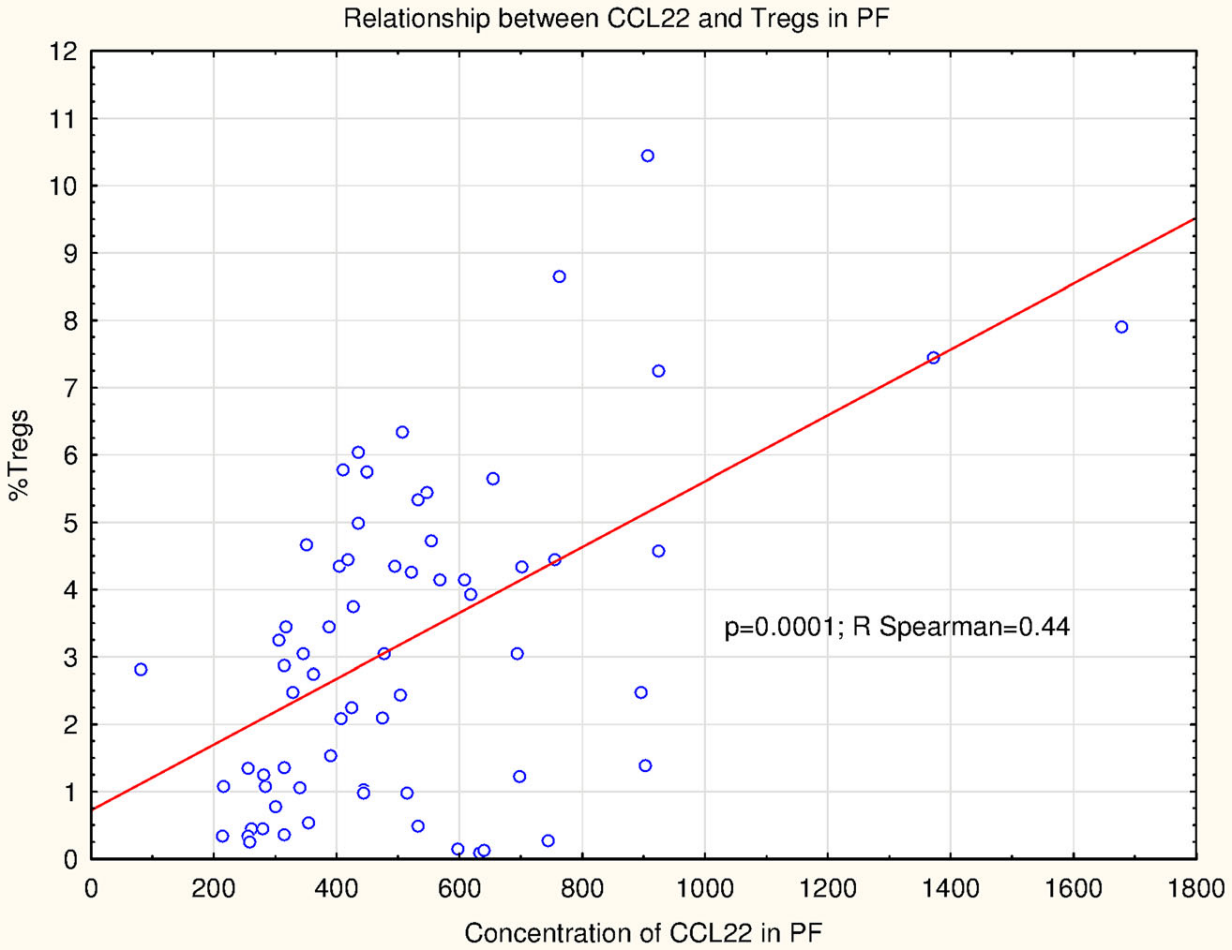
Correlations between CCL22 and Tregs in patients with ovarian cancer

The percentage of CD4^+^CD25^high^FoxP3^+^ cells positively correlated with the concentration of peritoneal fluid CCL22 levels (RSpearman = 0.44; *p* = 0.0001) (Fig. 4).There was no statistically significant correlations between the plasma CCL22 levels and Tregs in PB (*R* = −0.12; *p* = 0.81).

### The percentage of Tregs expressing CCR4

**Fig. 5:**
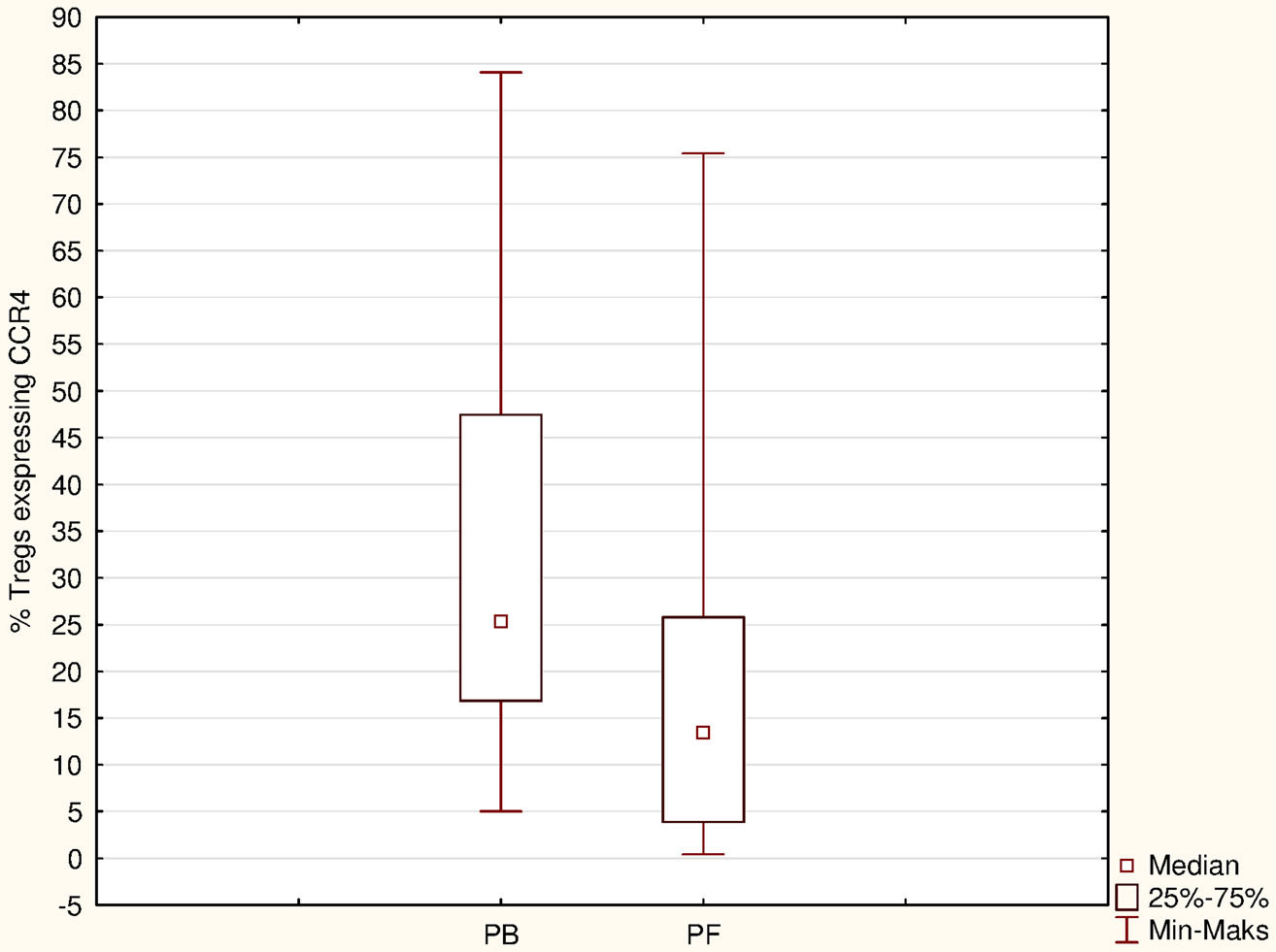
The percentage of Tregs expressing CCR4 in the PB and PF of women with ovarian cancer

The percentage of CD4^+^CD25^high^FoxP3^+^ cells expressing CCR4 receptor in the PB was slightly higher than in the PF, but no significant differences (*p* = 0.06) were observed (Fig. 5).

## Discussion

The results of the present study show for the first time positive relationship between triple stained CD4^+^CD25^high^FoxP3^+^ cells and concentration of CCL22 chemokine in the peritoneal fluid of patients with ovarian cancer. It is, therefore, tempting to speculate that Tregs accumulation in the peritoneal cavity of EOC patients is related to their chemotaxis from peripheral blood. It was also interesting whether changes in the concentration of CCL22 and the percentage of Tregs may depend on the stage, grade, and histological type of the tumor. To our knowledge, CCL22 levels and frequency of triple stained CD4^+^CD25^high^FoxP3^+^ cells have not been studied so far in EOC patients with different stage, grade, and histological type of the tumor.

We found concentrations of CCL22 in the peritoneal fluid to be significantly elevated in women with EOC, as compared to benign ovarian tumor patients. Additionally, the CCL22 levels in the peritoneal fluid of EOC patients were significantly higher than the plasma levels. Our results suggest that macrophage-derived chemokine, CCL22, may be produced locally by ovarian tumor cells. This hypothesis is consistent with the results of Curiel et al. [1] who clearly demonstrated expression of CCL22 mRNA in tumor tissue and ascites cells of EOC patients, and no such evidence in PBMC or normal ovaries. The authors also detected large amounts of CCL22 in tumor ascites, but only in 18 patients with EOC. However, no CCL22 concentration in the PF of women with benign ovarian tumors was assessed. Interestingly, our data suggest that malignant and benign tumors induce significantly different levels of CCL22 secretion.

The other studies showed that myeloid dendritic cells may be the source of CCL22 [11, 1, 12, 13]. Our previous studies have demonstrated that the percentage of myeloid DC in mononuclear cells was significantly higher in the PF than in PB of ovarian cancer patients [27]. However, Goyne et al. [28] recently proved that primary tumor ascites CD14^+^ cells, rather than tumor cells, were the predominant source of CCL22 in a cohort of nine patients with EOC.

The results of our group showed that the tissue and PF environment in women with malignant ovarian tumors contains considerably more Tregs than PB. This is in agreement with the results of previously published studies by Li et al. [29], who showed slightly increased Tregs percentage in the tumor tissue compared with PB and PF but the results did not achieve conventional level of statistical significance. Similarly to our results, Curiel et al. [1] reported significantly higher percentage of Tregs in the ascites than PB of ovarian cancer patients. The authors identified Tregs using flow cytometry by the expression of CD4^+^CD25^+^ antigens. In our study, we phenotyped Tregs as triple stained CD4^+^CD25^high^FoxP3^+^ cells. It should be emphasized that intracellular FoxP3 expression is nowadays the best marker available for Tregs [3]. Our observation appears to support the hypothesis that peripheral blood Tregs are specifically recruited into PF and cancer tissue. The reason for the accumulation of Tregs in the PF and cancer tissue has not been clearly explained yet.

Studies by Curiel et al. [1] suggest that CCL22 chemokine mediates Tregs trafficking in vitro and may recruit Tregs to the tumor. Moreover, the authors have shown that tumor Tregs express functional CCR4, the receptor for CCL22, and can migrate to CCL22, present in the tumor microenvironment. Interestingly, according to our data, the highest concentrations of CCL22 and the highest percentage of Tregs were found in the PF of women with EOC. Consistently with previous results [8, 1], our data showed that percentage of triple stained CD4^+^CD25^high^FoxP3^+^ cells expressed CCR4 receptor. Moreover, the percentage of CD4^+^CD25^high^FoxP3^+^ cells positively correlated with the concentration of PF CCL22 levels. This raises the possibility that high ascites CCL22 levels detected in our study may be a potent chemoattractant for Tregs expressing CCR4. It is worth noting that, in patients with serous cystadenoma, in contrary to patients with EOC, levels of CCL22 were consistently higher in the plasma than in PF. It has been also reported that Tregs can be converted from non-Tregs by high levels of TGF-β [30, 31, 16]. The previous study in hepatocellular carcinoma provided direct evidence that TGF-β could suppress the expression of miR-34a, resulting in the enhanced production of CCL22 and recruitment of Tregs. The authors speculate that Tregs accumulation may be induced through the interaction of TGF-β and CCL22 [32].

In the following study, we have carefully analyzed the concentration of CCL22 in the PF and plasma of women with different stages, grades, and histological types of tumor. We demonstrated that the plasma CCL22 concentrations are the highest in patients with FIGO stage IV of ovarian cancer. The peritoneal fluid CCL22 levels were the highest in patients with FIGO stage I of EOC. There were also significant differences in the PF CCL22 levels based on tumor histology. Unexpectedly, the highest concentrations of CCL22 were found in the PF of women with endometrioid cystadenocarcinoma and in plasma of women with mucinous cystadenocarcinoma. These data led us to conclude that CCL22 production in ovarian cancer patients depends on the stage and histological type of the tumor cells.

The results of our study suggest that increased level of CCL22 in the PF of women suffering from EOC may have an important role in migration of Tregs to the peritoneal cavity and thus may be responsible for suppression of local immune responses.
